# Supplementary material for: Causal Relationship between Aging and Anorexia Nervosa: A White-Matter-Microstructure-Mediated Mendelian Randomization Analysis
Source: Biomedicines. 2024 Aug 16;12(8):1874. doi: 10.3390/biomedicines12081874 (PMC11351342; doi:10.3390/biomedicines12081874)
Supplement: Supplementary file 1 [file biomedicines-12-01874-s001.zip › Supplementary Figure/Figure S8/Leaveoneout plot of MR analysis for TL to ubm-b-1548.pdf]

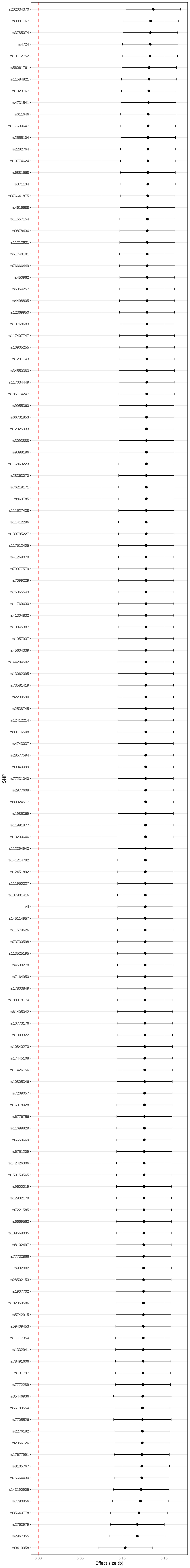
rs202034370
rs3891167
rs3785074
rs4724
rs10112752
rs56061761
rs11584821
rs1023767
rs4731541
rs611646
rs117630647
rs2555104
rs2282764
rs10774624
rs6881568
rs871134
rs376641875
rs4616688
rs11557154
rs9878436
rs11212631
rs61748181
rs76666449
rs450962
rs6054257
rs4498805
rs12369950
rs10768683
rs117407747
rs10905255
rs1291143
rs34550383
rs117034449
rs185174247
rs9955360
rs66731853
rs12925933
rs3093888
rs9398196
rs116863223
rs28363070
rs76219171
rs869785
rs111527438
rs11412296
rs139795227
rs117512405
rs41269079
rs79977579
rs7099229
rs76065543
rs11769630
rs41304832
rs10845387
rs1957937
rs45604339
rs144204502
rs13062095
rs73581419
rs2230590
rs2538745
rs12412214
rs80116508
rs4743037
rs28577594
rs9940099
rs77231040
rs2977608
rs80324517
rs1985369
rs11991877
rs13230646
rs112394943
rs141214782
rs12451892
rs111950327
rs137901416
A8
rs145114957
rs11579626
rs73730598
rs113525195
rs4530278
rs7164950
rs17803849
rs188918174
rs61405042
rs10773176
rs1003322
rs10840270
rs17445108
rs11426156
rs10805346
rs7209057
rs16978028
rs6776756
rs11699829
rs6659669
rs6751209
rs142426306
rs150150565
rs9600019
rs12932179
rs7221585
rs6669563
rs139669835
rs8102497
rs77732866
rs932002
rs28502153
rs1907702
rs182059586
rs5742915
rs59409453
rs11117354
rs1332941
rs78491606
rs131797
rs7772289
rs35446936
rs56799554
rs7705526
rs2276182
rs2056726
rs17677991
rs8105767
rs75664430
rs143190905
rs7790856
rs35640778
rs2763979
rs2967355
rs9419958
SNP
0.00
0.05
0.10
0.15
Effect size (b)
